# Patterns of CMR measured longitudinal strain and its association with late gadolinium enhancement in patients with cardiac amyloidosis and its mimics

**DOI:** 10.1186/s12968-017-0376-0

**Published:** 2017-08-07

**Authors:** Lynne K. Williams, Julian F. Forero, Zoran B. Popovic, Dermot Phelan, Diego Delgado, Harry Rakowski, Bernd J. Wintersperger, Paaladinesh Thavendiranathan

**Affiliations:** 1 Division of Cardiology, Peter Munk Cardiac Center, Ted Rogers Program in Cardiotoxicity Prevention, Toronto General Hospital, University Health Network, 200 Elizabeth Street, Toronto, ON M5G 2C4 Canada; 2 Department of Cardiology, Papworth Hospital NHS Foundation Trust, Cambridge, UK; 3 Department of Radiology and Diagnostic Imaging, Center for Excellence in Cardiovascualr Imaging, Fundación Cardioinfantil Instituto de Cardiología, Bogotá, Colombia; 4 Department of Medical Imaging, University Health Network, Toronto, Canada; 5 Heart and Vascular Institute, Cleveland Clinic, Cleveland, OH USA

**Keywords:** Cardiovascular magnetic resonance imaging, Left ventricular hypertrophy, Late gadolinium enhancement, Myocardial strain, Cardiac amyloidosis, Anderson Fabry’s disease, Hypertrophic cardiomyopathy

## Abstract

**Background:**

Regional variability of longitudinal strain (LS) has been previously described with echocardiography in patients with cardiac amyloidosis (CA), however, the reason for this variability is not completely evident. We sought to describe regional patterns in LS using feature-tracking software applied to cardiovascular magnetic resonance (CMR) cine images in patients with CA, hypertrophic cardiomyopathy (HCM), and Anderson-Fabry’s disease (AFD) and to relate these patterns to the distribution of late gadolinium enhancement (LGE).

**Methods:**

Patients with CA (*n* = 45) were compared to LV mass indexed matched patients with HCM (*n* = 19) and AFD (*n* = 19). Peak systolic LS measurements were obtained using Velocity Vector Imaging (VVI) software on CMR cine images. A relative regional LS ratio (RRSR) was calculated as the ratio of the average of the apical segmental LS divided by the sum of the average basal and mid-ventricular segmental LS. LGE was quantified for the basal, mid, and apical segments using a threshold of 5SD above remote myocardium. A regional LGE ratio was calculated similar to RRSR.

**Results:**

Patients with CA had significantly had worse global LS (−15.7 ± 4.6%) than those with HCM (−18.0 ± 4.6%, *p* = 0.046) and AFD (−21.9 ± 5.1%, *p* < 0.001). The RRSR was higher in patients with CA (1.00 ± 0.31) than in AFD (0.79 ± 0.24; *p* = 0.018) but not HCM (0.84 ± 0.32; *p* = 0.114). In CA, a regional difference in LGE burden was noted, with lower LGE in the apex (31.5 ± 19.1%) compared to the mid (38.2 ± 19.0%) and basal (53.7 ± 22.7%; *p* < 0.001 for both) segments. The regional LGE ratio was not significantly different between patients with CA (0.33 ± 0.15) and AFD (0.47 ± 0.58; *p* = 0.14) but lower compared to those with HCM (0.72 ± 0.43; *p* < 0.0001). LGE percentage showed a significant impact on LS (*p* < 0.0001), with a 0.9% decrease in absolute LS for every 10% increase in LGE percentage.

**Conclusion:**

The presence of marked “relative apical sparing” of LS along with a significant reduction in global LS seen in patients with CA on CMR cine analysis may provide an additional tool to differentiate CA from other cause of LVH. The concomitant presence of a base to apex gradient in quantitative LGE burden suggests that the regional strain gradient may be at least partially explained by the burden of amyloid deposition and fibrosis.

## Background

Cardiac involvement in amyloidosis is a major determinant of adverse outcomes [1]. In addition, the prevalence of cardiac amyloidosis in patients over the age of 65 years with clinical symptoms of heart failure with a preserved ejection fraction has been demonstrated to be as high as 29% in a recent study [2]. Clinical history, electrocardiograms, echocardiography, nuclear imaging, and cardiovascular magnetic resonance (CMR) play a complementary role in the diagnosis of cardiac amyloidosis (CA). With CMR, classic morphologic features, characteristic difficulty “nulling” the myocardium on post gadolinium images, and diffuse late gadolinium enhancement (LGE) in non-ischemic patterns, are traditionally used to establish the diagnosis [3]. Patterns of LGE seen in CA have been related to the deposition of amyloid protein and/or myocardial fibrosis [3]. However, it can often be challenging to obtain diagnostic quality LGE imaging in these patients due to difficulty in identifying the correct myocardial nulling time. Furthermore, approximately one third of patients with systemic amyloidosis have evidence of renal dysfunction [4] precluding the acquisition of LGE imaging.

Recent echocardiography-based studies have demonstrated a characteristic pattern of regional longitudinal strain (LS) abnormalities that can differentiate CA from other conditions associated left ventricular hypertrophy (LVH) [5]. This pattern referred to as “relative apical sparing” describes a reduction in LS in the basal and mid-myocardial segments, with relative sparing of the left ventricular apex. This pattern has been associated with increased accuracy for the diagnosis of CA and subsequent prognosis [6, 7]. However, echocardiography can pose a challenge in patients with difficult acoustic windows, particularly with regards to imaging of the true left ventricular apex. In these patients CMR presents a helpful alternative for imaging the entire left and right ventricular myocardium. More recently, feature tracking software has been applied to cine CMR datasets for assessment of left ventricular strain, with good agreement between CMR and 2D echocardiography-derived myocardial LS measurements [8]. In patients with undifferentiated LVH, the ability to perform LS analysis on CMR cine images to demonstrate this pattern of “relative apical sparing” may present a useful adjunct in the identification of CA, particularly in patients with suboptimal echocardiographic image quality. In the current study we hypothesized that: (1) LS measurements using a feature tracking software on cine CMR images would allow identification of a “relative apical sparing” pattern in patients with CA, and that would allow differentiation from other conditions associated with increased LV mass and (2) that the regional left ventricular LS variations seen in patients with CA are related to variations in the relative distribution of LGE on CMR images.

## Methods

### Patients

This was a retrospective study of all patients referred to the Department of Medical Imaging at the Toronto General Hospital for CMR imaging between 2003 and 2013 who were diagnosed with CA. The indication for CMR included assessment of LVH identified by other imaging modalities, or to detect cardiac involvement in patients with a known diagnosis of systemic amyloidosis. Medical chart review of all patients was performed. Patients with a confirmed diagnosis of CA were included. The diagnosis of CA was confirmed either by endomyocardial biopsy consistent with CA [9] or by extra-cardiac biopsy confirming systemic amyloidosis and CMR features consistent with cardiac involvement [10]. Exclusion criteria included significant arrhythmia resulting in CMR image degradation, CMR cine images unsuitable for feature tracking analysis, non-diagnostic LGE images, or insufficient clinical information to allow confirmation of a diagnosis of CA.

As comparator groups, a random sample of patients with non-obstructive hypertrophic cardiomyopathy (HCM) and Anderson-Fabry’s disease (AFD) who were referred for CMR, with similar overall left ventricular mass index to the CA group, who had balanced steady state free precession (SSFP) cine images suitable for feature tracking analysis and diagnostic quality LGE images were included. These comparator groups were chosen as these two diseases are in the differential diagnosis of patients presenting to the CMR lab with undifferentiated LVH. All patients with HCM were under active follow-up in the Hypertrophic Cardiomyopathy Clinic at the Toronto General Hospital. The diagnosis was based on the finding of unexplained LVH with a maximal wall thickness ≥ 15 mm in any myocardial segment [11]. Patients with significant resting or provocable left ventricular outflow tract obstruction were excluded, as were patients with apical HCM (due to the confounding effects of these factors of both global and regional strain parameters). All patients with AFD had a confirmed diagnosis based on enzyme testing/genetic testing and were under active follow-up in the Metabolic Disease Clinic at University Health Network.

### Cardiac magnetic resonance imaging

All CMR studies were performed on one of the following systems: 1.5 T Sigma HDx Twin Speed (General Electric Healthcare, Waukesha, WI, USA), a 1.5 T Magnetom Avanto (Siemens Healthcare, Erlangen, Germany), or a 3.0 T Magnetom Verio (Siemens Healthcare, Erlangen, Germany) equipped with either an 8 or 32-element phased-array coil. The following imaging sequences were performed: (1) a short-axis stack and long axis (2,3, 4 chamber) retrospectively-gated cine steady state free precession (SSFP) images of the left ventricle, (2) inversion recovery (IR) gradient-recalled echo (GRE) or SSFP for LGE imaging with identical ventricular coverage of the LV as for short axis cine. Typical SSFP parameters were as follows: spatial resolution of 1.3–1.5 × 1.3–1.5 mm, slice thickness 6-8 mm with a gap of 0-2 mm, and a temporal resolution of 35-50 msec. LGE imaging was performed 7–10 min after the administration of 0.2 mmol/kg gadobutrol (Gadovist, Bayer Healthcare, Berlin, Germany) using TI scout for contrast optimization. Both magnitude and phase sensitive inversion recovery (PSIR) images were acquired.

Careful assessment was performed to ensure adequate quality of images used for analysis and comparable protocols. Adequacy of the cine images was defined as absence of arrhythmia artefact to allow adequate tracking by VVI. For LGE imaging adequacy was determined as a selected inversion time that allowed differentiation between enhanced and non-enhanced myocardium, as well as verification against the T1 scout sequences and PSIR sequences.

### Myocardial global longitudinal systolic strain analysis

VVI is an angle-independent feature-tracking method which incorporates feature and endo- and epicardial contour tracking. The feature-tracking program, VVI Version 3.0.0 (Siemens Healthcare, Mountain View, CA), was applied to CMR cine SSFP images from archived studies, allowing peak systolic longitudinal strain (LS) measurement as previously described by our group [8]. Myocardial motion was quantified by automatic tracking of user-defined points in subendocardial regions in order to derive LS. Strain analysis was performed by an experienced independent observer (LW) who was blinded to the underlying diagnosis using randomized and de-identified images.

**Fig. 1:**
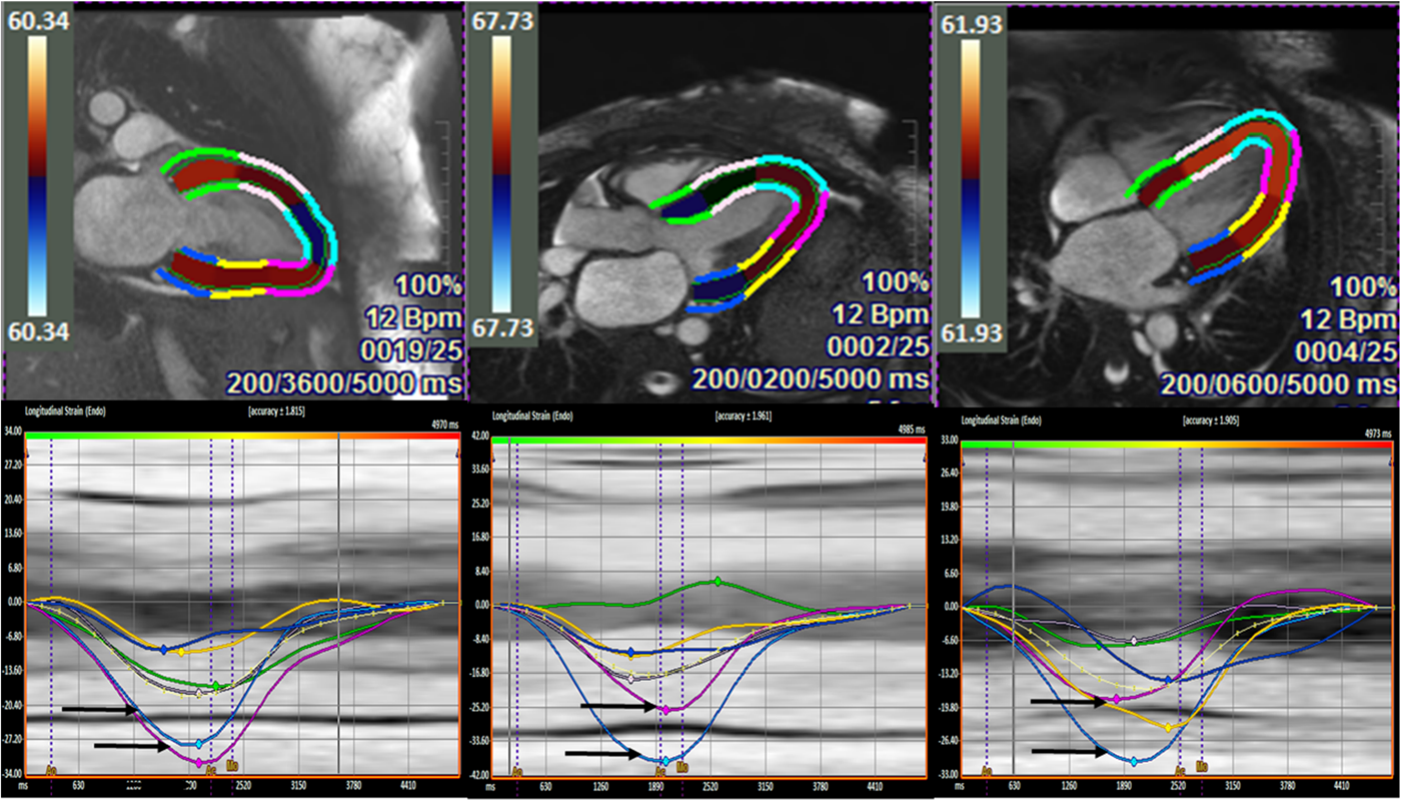
Representative examples of VVI strain analysis and curves in the 2, 3, and 4-chamber long axis views in a patient with cardiac amyloidosis. The *lower panel* demonstrates *regional strain curves*, with the *coloured curves* corresponding to the segments in the *upper panel*. In each of the views, the basal segments are represented by the *blue* and *green curves*, the mid-ventricular segments by the *white* and *yellow curves*, and the apical segments by the *turquoise* and *pink curves*. Note the highest regional strain values are consistently seen in the apical segments (highlighted by the *black arrows*) in all three long-axis views

Longitudinal strain was measured from the 4-chamber, 2-chamber, and 3-chamber CMR cine images. For each view, endocardial borders were manually traced in the end-diastolic frame. LS values were recorded after visual confirmation of best endocardial motion tracking (Fig. 1). Segments with poor tracking were excluded from further analysis. If more than two segments demonstrated poor tracking, the view was excluded from further analysis. Global LS values were obtained by averaging the segmental strain values (6 segments in each of the 4-chamber, 2-chamber and 3-chamber views for a total of 18 segments when data from all segments were available). Strain values for the six basal, six mid, and six apical segments of the LV were averaged to obtain three “regional” longitudinal strain values (averaged basal, averaged mid, and averaged apical strain values). The apex-to-base gradient in regional LS was examined using absolute strain values as well as a relative regional LS ratio (RRSR) which was calculated as follows: Average Apical LS/(Average basal LS + Average mid LS).

### CMR function and LGE quantification

**Fig. 2:**
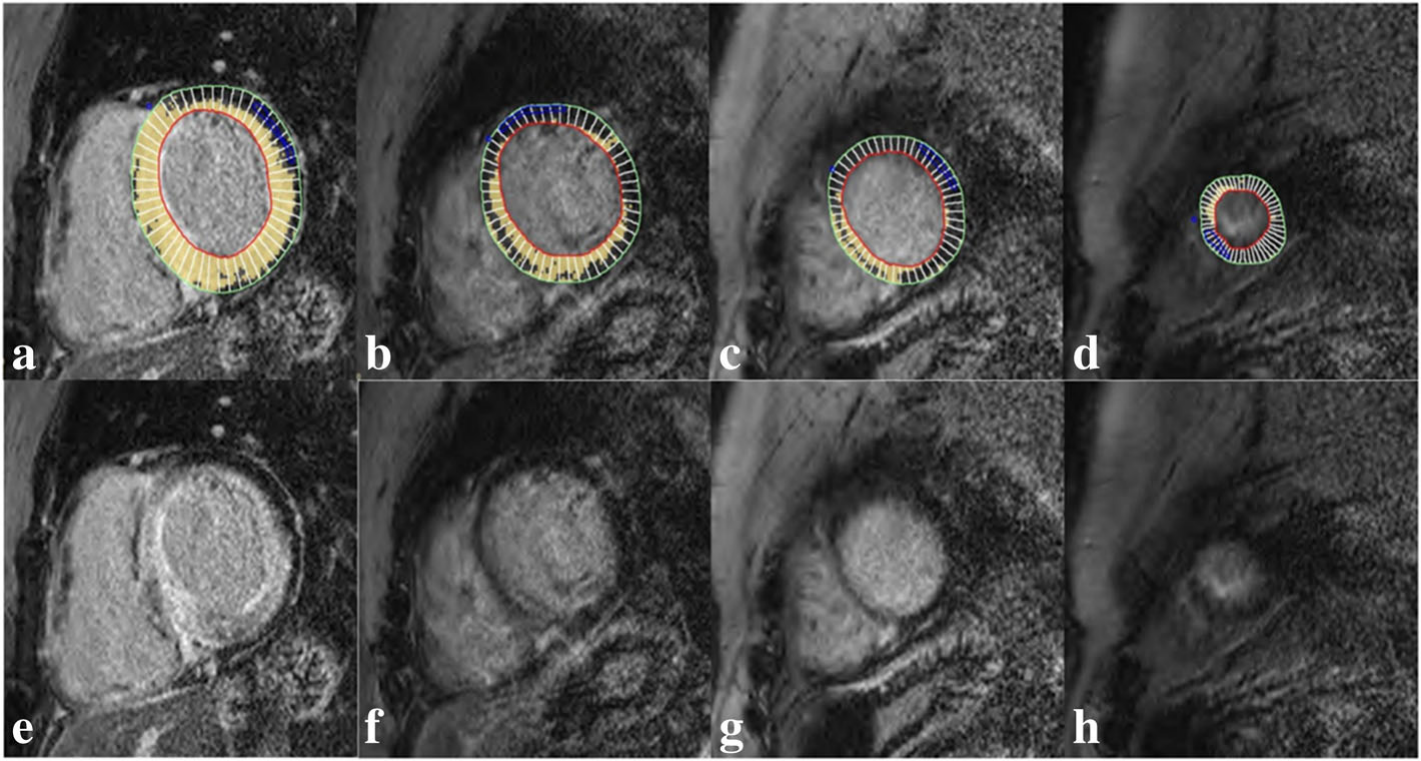
Late gadolinium enhancement (LGE) quantification. Comparison between the contoured (**a**-**d**) and source (**e**-**h**) LGE images in the same slice position to illustrate the quantification method and the resulting difference in the burden of LGE from the basal (**a** and **e**) towards the apical segments (**d** and **h**). For each of the contoured slices (**a**-**d**) the endocardial (*red*) and epicardial contours (*green*) as well as the reference area of non-enhanced myocardium (*blue*) have been defined

LV ejection fraction, volumes, mass and LV wall thickness for each of the 16 myocardial segments (as per American Heart Association segmentation) were quantified for all patients using commercially available software (cmr42, Circle Cardiovascular Imaging, Calgary, Canada) by a level 3 trained CMR reader, blinded to the underlying diagnosis of each patient. Mean wall thickness for the basal, mid, and apical LV segments were calculated by averaging the appropriate segmental values. For LGE quantification, a gray-scale threshold of 5 SD above the mean signal intensity of myocardium that demonstrated the lowest signal intensity (effectively nulled) was used (Fig. 2). Once LGE was identified automatically, the region of interest was visually confirmed and adjusted to ensure that enhancing myocardial segments were identified appropriately while non-enhancing segments were excluded. Values were reported as a percentage of area of enhancement of the LV and averaged for the basal, mid, and apical segments (Fig. 2). The total enhanced mass was calculated. A relative regional LGE ratio was calculated similar to the RRSR as: Average apical LGE/(Average basal LGE + Average mid LGE) using percentage enhancement for each ventricular level.

### Inter and Intraobserver variability

For intraobserver variability of global LS, RSSR, total enhanced mass of the LV, and the relative regional LGE ratio, 10 randomly selected patients with CA were re-analysed by the same observer, and for inter-observer variability, the same patients were analyzed by a second observer blinded to all previous measurements.

### Statistical analysis

All data were examined for normality by examining the kurtosis and skewness of the distribution. The Kolmogorov-Smirnov test was also used to asses for normality. Based on the distribution, data are presented as mean ± SD or median and 25-75^th^ percentiles for continuous data, while categorical data are presented as frequencies. We compared demographic data between the CA group versus the HCM and AFD groups using the Mann-Whitney Rank Sum Test for continuous data and Fisher’s exact test for categorical data.

Global and regional strain, strain ratio, LGE, and LGE ratio were compared between patients with CA and the other 2 groups using Mann-Whitney Rank Sum Test or Wilcoxon Test as appropriate. To assess the impact of LGE percentage, disease type, and left ventricular level on LS measurements, General Linear Measures analysis was performed using disease type (CA, HCM, AFD) as a fixed variable, left ventricular level (base, mid, or apex) and LGE percent as covariates, and patient ID as a random dummy variable. The sensitivity and specificity of the RSSR, relative LGE ratio, and a ratio of strain to LGE ratio for each ventricular level for the diagnosis of CA was assessed using receiver operator characteristic (ROC) curves. Intra- and inter-observer variability was assessed using calculation of intraclass correlation coefficients with 95% confidence intervals. A level of significance of 0.05 was used. All statistical analysis was performed using MedCalc version 11.6.0.0 (MedCalc Software, Belgium) or SPSS version 20 (SPSS Inc., International Business Machines, Chicago, Illinois, USA).

## Results

### Patient population

From an initial group of 51 patients with confirmed CA 6 patients were excluded due to poor endocardial tracking, resulting in a final study population of 45 patients. We identified 19 patients with HCM, and 19 with AFD who had images that met the quality standards required for analysis and had LV mass index in the same range as those with CA during the same period of recruitment for the CA patients. Patient characteristics are summarized in Table 1. There was no difference in the prevalence of HTN between the 3 groups. All patients were in sinus rhythm at the time of CMR.

**Table 1:** Baseline clinical and CMR characteristics

	CA (*n* = 45)	HCM (*n* = 19)	*p* value (vs CA)	AFD (*n* = 19)	*p* value (vs CA)
Age (years)	66.6 ± 10.0	57.0 ± 10.1	0.0004	49.3 ± 8.8	<0.0001
Sex (%male)	28 (62%)	14 (74%)	0.39	13 (68%)	0.65
Hypertension(%)	17 (38%)	7 (37%)	0.99	8 (42%)	0.78
ΒSA (m^2^)	1.85 ± 0.27	1.94 ± 0.21	0.17	1.92 ± 0.17	0.23
Heart rate at CMR (bpm)	76 ± 16	65 ± 17	0.009	65 ± 11	0.007
LVEF (%)	53.8 ± 11.2	61.6 ± 9.4	0.009	61.1 ± 6.4	0.009
LVEDVi (ml/m^2^)	80.9 ± 23.8	80.1 ± 17.0	0.86	90.4 ± 18.0	0.09
LVESVi (ml/m^2^)	39.1 ± 19.5	34.9 ± 18.5	0.46	34.9 ± 7.5	0.98
LVMI (g/m^2^)	90.5 ± 30.9	89.5 ± 28.8	0.98	87.2 ± 32.5	0.58
LV wall thickness basal LV (mm)	13.4 ± 2.8	12.5 ± 2.5	0.10	11.2 ± 3.0	0.004
LV wall thickness mid LV (mm)	11.4 ± 2.5	12.3 ± 4.2	0.41	10.2 ± 8.9	0.009
LV wall thickness apical LV (mm)	8.9 ± 2.4	9.1 ± 3.1	0.83	8.9 ± 4.4	0.21

*BSA* body surface area, *NYHA* New York Heart Association, *LVEF* left ventricular ejection fraction, *LVMI* left ventricular mass index, *LVEDVI* left ventricular end-diastolic volume index, *LVESVi* left ventricular end-systolic volume index, data presented as mean ± SD

In the CA group the diagnosis was confirmed by endomyocardial biopsy in 19 patients (42%), and by extra-cardiac biopsy confirmation of systemic amyloidosis with CMR features consistent with cardiac involvement in the remaining 26 patients (58%). The majority of patients had a diagnosis of AL amyloid (*n* = 33; 73%), with TTR amyloid in 11 patients (24%) and AA amyloid in 1 patient (2%).

### General CMR findings

Patient were well matched for left ventricular mass index, with no significant difference noted between those patients with CA (90.5 ± 30.9 g/m^2^) and the groups with HCM (89.5 ± 28.8; *p* = 0.98) and AFD (87.2 ± 32.5; *p* = 0.58). In addition, no significant differences were noted between groups in left ventricular end-diastolic or end-systolic volumes. The LVEF was significantly lower in patients with CA (53.8 ± 11.2%) compared to patients with HCM (61.6 ± 9.4%; *p* = 0.009) and AFD (61.1 ± 6.4%; *p* = 0.009).

### Global longitudinal strain (GLS)

**Fig. 3:**
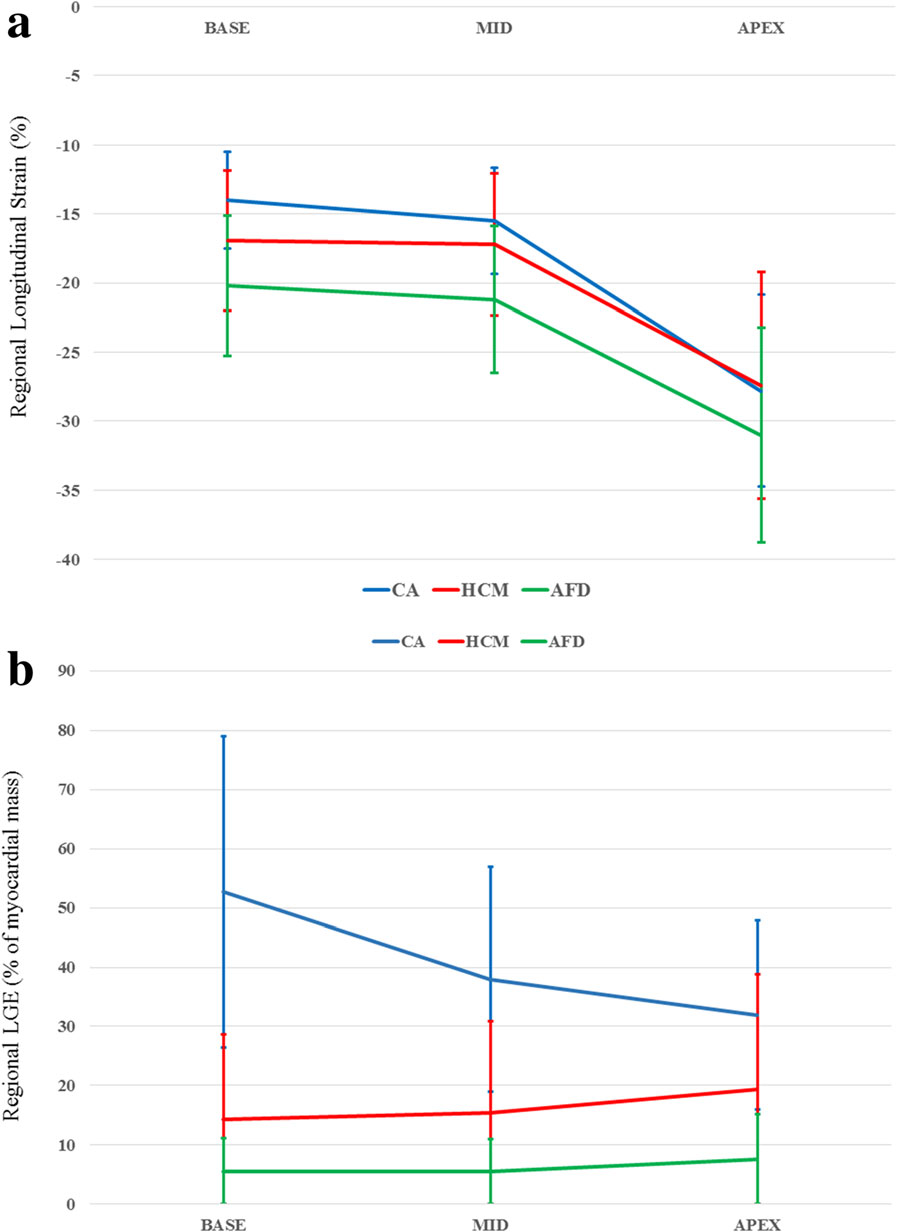
Comparison of basal to apical longitudinal strain values (*upper panel*) and late gadolinium enhancement as percentage of myocardial mass (*lower panel*) in the three disease groups. A larger relative difference in basal to apical longitudinal strain and late gadolinium enhancement are seen in patients with cardiac amyloidosis (CA) compared to hypertrophic cardiomyopathy (HCM) and Anderson-Fabry’s disease (AFD)

Peak systolic GLS was significantly decreased in patients with CA compared to those with HCM (−15.7 ± 4.6% vs −18.0 ± 4.6%; *p* = 0.046) and AFD (−15.7 ± 4.6% vs −21.9 ± 5.1%; *p* < 0.001). A basal to apical strain gradient was seen in all three disease states, with the highest absolute strain values seen in the apical segments (as demonstrated in Table 2 and Fig. 3). This gradient was most prominent in patients with CA. In these patients, LS was significantly higher in the apical level (−27.3 ± 7.5%) compared to either the basal (−14.3 ± 5.9%) or mid segments (−15.5 ± 6.3%), *p* < 0.001 for both. This was reflected in the RRSR which was higher in patients with CA (1.00 ± 0.31) than in patients with HCM (0.84 ± 0.32) and AFD (0.79 ± 0.24). But a statistically significant difference in RSSR was only seen between CA and AFD (*p* = 0.018) but not between CA and HCM (*p* = 0.114). Using receiver operator characteristics (ROC) analysis, RSSR had an AUC of 0.66 (95% CI: 0.55–0.76) for the diagnosis of CA with a RRSR of >1.05 providing a specificity of 82% but a sensitivity of only 43% in differentiating CA from other causes of LVH.

**Table 2:** Longitudinal strain and late gadolinium enhancement parameters

	CA (*n* = 45)	HCM (*n* = 19)	*p* value (vs CA)	AFD (*n* = 19)	*p* value (vs CA)
Longitudinal strain					
Average basal LS (%)	−14.3 ± 5.9	−16.9 ± 4.9	0.049	−20.2 ± 5.4	<0.001
Average mid LS (%)	−15.5 ± 6.3	−17.2 ± 4.5	0.128	−21.1 ± 5.3	0.002
Average apical LS (%)	−27.3 ± 7.5	−27.4 ± 9.7	0.959	−31.0 ± 5.7	0.082
Average Global LS (%)	−15.7 ± 4.6	−18.0 ± 4.6	0.046	−21.9 ± 5.1	<0.001
Late Gadolinium Enhancement					
Average basal LGE (%)	53.7 ± 22.7	13.6 ± 14.6	<0.001	5.4 ± 4.9	<0.001
Average mid LGE (%)	38.2 ± 19.0	14.9 ± 19.7	<0.001	5.4 ± 5.6	<0.001
Average apical LGE (%)	31.5 ± 19.1	19.7 ± 25.1	0.005	6.6 ± 10.9	<0.001
Average total LGE (%)	50.6 ± 25.5	17.3 ± 20.2	<0.001	6.6 ± 7.2	<0.001

*LS* longitudinal strain, *LGE* late gadolinium enhancement

### Late gadolinium enhancement

There was a difference in total LGE burden between the groups, with the largest amount of LGE seen in patients with CA (50.6 ± 25.5%) compared to HCM (17.3 ± 20.2%; *p* < 0.001) and AFD (6.6 ± 7.2%; *p* < 0.001). There were also differences in LGE burden at the different ventricular levels (base, mid, and apex) as demonstrated in Table 2. LGE percentage was significantly affected by disease aetiology (*p* < 0.001) and left ventricular level (*p* < 0.01). There was a significant interaction within the CA group between left ventricular level and LGE burden, with a 14% increase in LGE for every increase in level from apex to base (*p* < 0.001). Such a relationship was not seen in either AFD or HCM. Differences in LGE distribution between the three disease groups are summarized in Table 2 and demonstrated in Fig. 3. The LGE ratio was lower in CA (0.33 ± 0.15) compared to HCM (0.72 ± 0.43, *p* < 0.001) but not AFD (0.47 ± 0.58, *p* = 0.562). In HCM and AFD there was a slight progressive increase in LGE from the base to the apex of the ventricle while in patients with CA there was a decrease (Fig. 3b). Patients with CA with an RRSR >1.05 had a lower mean LGE ratio at 0.28 compared to those values ≤1.05 where the LGE ratio was 0.38 (*p* = 0.018).

### Impact of LGE on GLS

In these groups of subjects with increased LV mass, LGE percentage showed a small but significant impact on global longitudinal strain (GLS) (*p* < 0.0001), with a 0.9% decrease in absolute GLS for every 10% increase in LGE percentage. There was also a weak but statistically significant correlation between LGE ratio and strain ratio for the entire group (*r* = −0.28, *p* = 0.011).

### Impact of amyloid subtype

There were no statistically significant differences in GLS (−15.9 ± 4.1% versus −14.9 ± 6.0%, *p* = 0.604), RSSR (0.99 ± 0.31 versus 1.01 ± 0.33, *p* = 0.902), or relative LGE ratio (0.35 ± 0.14 versus 0.29 ± 0.15, *p* = 0.263) between those with AL and TTR amyloidosis respectively.

### Discriminatory value of LS and LGE

Using receiver operator characteristics (ROC) analysis, RSSR had an AUC of 0.66 (95% CI: 0.55–0.76) for the diagnosis of CA with a RRSR of >1.05 providing a specificity of 82% but a sensitivity of only 43% in differentiating CA from other causes of LVH. The discriminatory value of other parameters including strain to LGE ratios are summarized in Table 3. When LGE data was combined with strain data the ratio of the basal LS to the basal LGE percentage had excellent discriminatory value for CA.

**Table 3:** The discriminatory value of various strain and LGE parameters to differentiate Cardiac Amyloidosis from Fabry’s Disease or Hypertrophic Cardiomyopathy

Ratio	AUC (95% CI)	Discrimination threshold	Sensitivity	specificity
RSSR	0.66 (0.55–0.76)	>1.05	43.0%	82.0%
LGE ratio alone	0.62 (0.51–0.72)	≤0.58	97.9%	44.7%
RSSR/Relative LGE ratio	0.67 (0.55–0.77)	>1.20	97.8%	44.4%
Basal strain/basal LGE^a^	0.94 (0.88–0.98)	≤0.77	91.1%	84.2%
Mid strain/mid LGE^a^	0.90 (0.81–0.96)	≤1.64	93.3%	79.0%
Apical strain/apical LGE^a^	0.80 (0.69–0.88)	≤2.80	86.7%	70.3%

^a^Calculated as the absolute value of the longitudinal strain for that ventricular level (i.e. base, mid, or apical) divided by the LGE percentage for that same level

### Inter and Intraobserver variability

Inter- and intra-observer variability for measurement of LS, RSSR, total LGE mass, and LGE ratio is summarized in Table 4. There was good reproducibility for all these measurements.

**Table 4:** Intra and interobserver variability of longitudinal strain and late gadolinium enhancement measurements

	Intraclass coefficient (95% CI)
Intraobserver	
GLS (%)	0.99 (0.95–1.00)
RSSR	0.96 (0.86–0.99)
Total LGE mass	0.97 (0.88–0.99)
LGE Ratio	0.87 (0.59–0.97)
Interobserver	
GLS (%)	0.98 (0.92–1.00)
RSSR	0.96 (0.86–0.99)
Total LGE mass	0.96 (0.85–0.99)
LGE Ratio	0.92 (0.75–0.98)

## Discussion

In the current study we demonstrate for the first time that a pattern of relative apical sparing of LS can be identified using CMR cine images in patients with CA. This pattern can be present in AFD and HCM as well, emphasizing the importance of calculating the RSSR to differentiate CA from other etiologies with increased LV mass. A calculated RSSR of >1.05 had a specificity of 82% for the diagnosis of CA. We also demonstrate a progressive 14% increase in quantitative regional myocardial LGE burden for each level progressing from the apex to the base in patients with CA only. For every 10% increase in LGE burden there was a 0.9% reduction in LS. Although this does not demonstrate a causal relationship, our data does suggest that the presence of apical sparing of LS in CA may represent the relatively lower deposition of amyloid protein and resultant fibrosis in the apical myocardial segments compared to the basal segments. Our work also demonstrates that when LS and LGE data are available in patients, the use of basal LS to LGE ratio provides excellent discrimination of CA from AFD and HCM.

A regional variation in distribution of LGE for patients with CA has been previously described in some subtypes of CA [12]. It is understood that the LGE seen in patients with CA relates to the deposition of amyloid fibrils [3, 13, 14]. A recent study assessing myocardial circumferential strain on CMR has demonstrated a significant reduction in patients with CA with LGE compared with those without LGE, suggesting a potential role for strain analysis for detection of LGE without the need for contrast medium [15]. A difference in ventricular function has also been demonstrated between the basal and apical segments of the left ventricle by echocardiography measured LS [5]. A relative apical sparing ratio calculated using echocardiography measured LS has been shown to have good specificity for the diagnosis of CA [5]. This regionality in LS has not been previously examined with CMR cine images. We demonstrate for the first time that the relative apical sparing of LS can also be identified with LS measurements performed on routinely acquired CMR cine images.

Despite the clinical use of echocardiography based LS in many laboratories to identify CA, the mechanism of this apical sparing has not been understood. In a recent study Ternacle et al. demonstrated a base to apical gradient in visually identified LGE by CMR and LS measured by echocardiography [16]. In 3 patients with myocardial tissue obtained at transplant they also demonstrated a variation in amyloid deposit from base to apex [16]. We extend these findings in the current study, by using CMR images both for LS measurements and quantitative LGE from the same study. In addition, we have compared LS and LGE measurements in patients with HCM and AFD which are phenocopies of CA. Our data supports the hypothesis that that the apical sparing pattern of LS in CA may be related to the differences in deposition of amyloid fibrils (as demonstrated by LGE) between the basal and apical myocardium. This differential distribution of amyloid deposition may result in adverse remodelling of the extracellular matrix, increased fibrosis, impaired contractility, and, in addition, may have direct mechanical effect on myocardial function or impact contractility through oxidative stress [17, 18]. Other mechanisms include the promotion of cardiomyocyte apoptosis by amyloid deposition contributing to regional functional differences [19].

Our work also demonstrates that other phenocopies of CA such as HCM and AFD can also have a basal to apical differences in LGE. The largest gradient in LGE from the apex to base (14% increase per level from apex to base) was seen in patients with CA similar to the gradient in strain. The presence of basal to apical gradient in LS in patients with HCM is similar to previous work with echocardiography [5]. The LS gradient seen in HCM and AFD in our study may be due to other mechanisms of myocardial dysfunction that are not accounted for by LGE imaging alone. For example in patients with HCM myocardial disarray and small intramural coronary artery disease can all contribute to myocardial dysfunction. This may account for the fact that despite lower amount of regional LGE differences in patients with HCM compared to CA (Table 2) there remained a reduction in regional LS at the basal and mid ventricular segments. In addition, there may also be a threshold of LGE for a reduction in strain beyond which additional LGE may not incrementally contribute to further reduction. Our work also highlights the fact that some patients with CA may not have apical sparing as demonstrated by low sensitivity of the RSSR ratio. Therefore, clinicians need to be cautious in using the absence of apical sparing to rule out CA. This is likely due to the fact that these patients without a large LS gradient have advanced disease with amyloid infiltration involving the apex as well. This was seen in our study as the LGE ratio was significantly higher in patients with CA and an RSSR ≤1.05 suggesting that the gradient in LGE may be less prominent.

Other than suggesting a potential mechanism for relative apical sparing of LS in CA, our current study has clinical relevance. A significant proportion of patients referred for the assessment of cardiac involvement by CMR in systemic amyloidosis have renal impairment prohibiting the use of gadolinium based contrast agents. Furthermore, non-diagnostic LGE images are common in these patients. Therefore, in addition to morphologic features, the finding of relative apical sparing by LS on routinely acquired cine SSFP images may aid with the diagnosis of CA. The finding of relative apical sparing in LS had good specificity for the diagnosis of cardiac amyloidosis in our study. The strength of this LS analysis is that no additional imaging is necessary and strain packages are now being incorporated into CMR analysis software making the measurement of LS possible during the clinical routine. Furthermore, in patients in whom gadolinium based contrast agents can be used, and diagnostic quality LGE data is available, a low ratio of regional LS to percentage LGE especially for base of the LV appears to be excellent in discriminating CA from HCM and AFD. This can be used as an additional tool in routine clinical practice.

## Limitations

This is a retrospective cohort study with a small number of patients and unequal patient population sizes. However, the study is focused on diseases with low prevalence including CA and AFD. Our sample size compares to previously published studies of strain variability in CA [5, 16]. Although we focused on two common causes of increased LV mass seen in the CMR laboratory, other diseases such as aortic stenosis and hypertensive heart disease can also present with LVH. However, the latter two diseases can be more readily identified based on other morphological findings and clinical history while the prior two are more challenging to differentiate from CA. Also our comparison groups were matched for LVMI and not for concentric hypertrophy, age, or LVEF. This reflects that fact that the lack of at least mild asymmetric hypertrophy even in AFD and HCM is not common, and that the latter diseases tend to occur in younger patients with a reduction in LVEF being a relative late stage of disease. The current study utilized VVI as the feature tracking software for assessment of myocardial strain, in contrast to the use of the GE EchoPac software in previous studies [5, 16]. However, VVI strain has been validated previously using echocardiography and CMR [8, 20]. We also did not have pathological confirmation of differences in amyloid deposition between the different myocardial segments. However, in conjunction with previous work [10, 16] that has shown that areas of LGE in patients with CA correspond to sites of amyloid deposition and fibrosis we can only hypothesize that strain abnormalities may relate to differences in amyloid deposition. Finally, over the study period we used different CMR systems for imaging. Whether the difference between these CMR systems has an impact on quantitative LGE and LV strain parameters is not certain. For LGE, our primary measure was the relative differences between slices in the same patient and in addition for each patient we ensured that the area of LGE identified by the post-processing software was consistent with the visual assessment of myocardial LGE extent. For LV strain, two recent publications assessing the effect of field strength on left ventricular volumes, LVEF, and strain parameters have shown no significant differences between 1.5 and 3.0 T field strength [21, 22].

## Conclusions

Our study demonstrates that regional differences in myocardial LS, can be identified by analysis of routinely acquired CMR cine images in patients with CA. Therefore, the phenomenon of relative apical sparing of LS in patients with CA is not only limited to echocardiographic strain analysis. (4) A RSSR ratio of >1.05 had good specificity for the diagnosis of CA when compared to other common causes of LVH. Also the fact that there was lower LGE burden in the apical myocardial segments compared to the basal segments especially in CA patients with an RSSR >1.05 suggests a potential association with amyloid deposition/fibrosis and the apical sparing in LS. Further studies using other quantitative CMR methods such as T1 mapping may help better determine the association between LS and the distribution of amyloid deposit/fibrosis in patients with CA. Finally, a regional ratio of LS and LGE may be an additional tool for the diagnosis of CA in routine practice when diagnostic quality LGE and cine images are available.

## Abbreviations

AFD: Anderson-Fabry’s Disease
CA: Cardiac amyloidosis
CMR: Cardiovascular magnetic resonance
GLS: Global longitudinal strain
GRE: Gradient-recalled echo
HCM: Hypertrophic Cardiomyopathy
HTN: Hypertension
IR: Inversion Recovery
LGE: Late gadolinium enhancement
LS: Longitudinal Strain
LVEF: Left ventricular ejection fraction
LVH: Left ventricular hypertrophy
LVMI: Left ventricular mass index
PSIR: Phase sensitive inversion recovery
ROC: Receiver operating characteristics
RSSR: Relative regional strain ratio
SSFP: Steady State Free Precession
VVI: Vector velocity imaging
